# Bio-butanol sorption performance on novel porous-carbon adsorbents from corncob prepared via hydrothermal carbonization and post-pyrolysis method

**DOI:** 10.1038/s41598-017-12062-7

**Published:** 2017-09-18

**Authors:** Mengjun Han, Kangkang Jiang, Pengfei Jiao, Yingchun Ji, Jingwei Zhou, Wei Zhuang, Yong Chen, Dong Liu, Chenjie Zhu, Xiaochun Chen, Hanjie Ying, Jinglan Wu

**Affiliations:** 1 College of Biotechnology and Pharmaceutical Engineering, Nanjing Tech University, Nanjing, China; 2 National Engineering Technique Research Center for Biotechnology, Nanjing, China; 3 Jiang su National Synergetic Innovation Center for Advanced Materials, Nanjing, China; 4 State Key Laboratory of Materials-Oriented Chemical Engineering, Nanjing, China

## Abstract

A series of porous-carbon adsorbents termed as HDPC (hydrochar-derived pyrolysis char) were prepared from corncob and used for the 1-butanol recovery from aqueous solution. The influences of pyrolysis temperature on properties of the adsorbents were systematically investigated. The results showed that hydrophobicity, surface area, and pore volume of HDPC samples increased with an increase in pyrolysis temperature. Furthermore, the adsorption mechanism of 1-butanol on the adsorbents was explored based on correlation of the samples properties with adsorption parameters extracted from the 1-butanol adsorption isotherms (*K*_*F*_ and *Q*_*e12*_). Overall, the 1-butanol adsorption capacity increased with a decrease in polarity and an increase in aromaticity, surface area and pore volume of HDPC samples. However, at different pyrolysis temperature, the factors causing the increase of 1-butanol adsorption on the adsorbents are variable. The kinetic experiments revealed that the pores played a vital role in the 1-butonal adsorption process. The intraparticle diffusion model was used to predict the adsorption kinetic process. The simulation results showed that intraparticle diffusion was the main rate-controlling step in the 1-butanol adsorption process.

## Introduction

Hydrochar is a charred material which is obtained by hydrothermal carbonization (HTC) of biomass at relatively low temperature (160–300 °C) in the presence of water under self-generated pressure^1,2^. As a novel carbonaceous material hydrochar has gained a great deal of attention because of its low production costs, environmental-friendly, and high value-added^3–5^. Recently, most studies in the literature mainly concentrated on the influence of hydrothermal conditions (temperature, time) and feedstock type (food residue, agriculture, etc) on the hydrochar production^6–9^. Its application is focused on heterogeneous catalysis, adsorption, soil enrichment and energy storage^10–12^. As adsorbents, hydrocahr has been used for adsorption of heavy metals and organic aromatic contaminants from environment^13,14^. Li *et al*. utilized bamboo hydrochars which produced at different hydrothermal conditions to adsorb hazardous substances from aqueous solutions, and demonstrate hydrochar can be used for potential adsorbent in waste water treatment^15^. The adsorption capacity for Congo red and 2-naphthol is 33.7 mg/g and 12.2 mg/g at the equilibrum concentration of 0.1 mg/mL, respectively. However, hydrochar as adsorbent has several shortcomings which greatly restrict its application. For instance, it contains various polar superficial function groups, which hinder adsorption of nonpolar organic matters. A negligible surface area and limited porosity also decrease the adsorption capacity of hydrochar, due to the insufficient binding sites to attach adsorbate^16,17^. Additionally, the low stability of hydrochar implies that it is not suitable as a long-term recycled adsorbent. Thus, a post-treatment method is required to overcome those problems.

Thermal treatment is regarded as one of the easiest post-treatment methods to adjust the properties of hydrochar^18^. Pyrolysis of hydrochar at relatively low temperature (<800 °C) under an inert atmosphere in a charred with excellent performance, termed hydrochar-derived pyrolysis char (HDPC)^19^. Different pyrolysis temperature significantly influence the characteristics of hydrochars^20^. Yu *et al*. have investigated how pyrolysis temperature effected on the properties of HTC materials, and the post-carbonized samples were used to selectively adsorb CO_2_ and N_2_^21^. Zhu *et al*. have also researched the effects of activation temperature on characterization of HDPC samples and on the performance of HDPC samples in adsorbing tetracycline from aqueous solutions. They demonstrated that the characteristics of HDPC samples generated at different carbonization temperatures determined the behavior or capacity of tetracycline adsorption^22^. The correlation between various HDPC samples and the adsorption thermodynamics parameters was established to explore how the HTC parameters (peak temperature, retention time, feedstock type) affect the properties of hydrochar and HDPC materials. However, there is no information related to study the adsorption kinetic and adsorption mechanism of adsorbate on HDPC materials.

It is well known that adsorption is affected by lots of factors, such as surface area, hydrophobicity/hydrophilicity and porosity structure of adsorbent^23^. In the process of preparing HDPC samples, the changes in pyrolysis temperature can control above mentioned factors. Thus, the use of hydrochar and HDPC materials as adsorbent, can more systematically reveal the adsorption behavior and mechanism of samples on the materials.

Biobutanol is considered as a promising alternative to petroleum-based chemicals, due to the depletion of fossil fuels and the increase of environmental problems^24–26^. However, a main challenge in commercial production of biobutanol is the separation and recovery of production from the dilute fermentation broth^27^. The energy consumption of 1-butanol recovery by traditional distillation is higher than the energy content of the product itself, especially when the concentration of the final product is low^28^. Among alternative separation techniques to remove 1-butanol from fermentation broth, chromatography separation based on adsorption has been identified as an attractive option^29^. In recent studies, a numbers of materials have been used for 1-butanol adsorption, including activated carbon, zeolites, ZIF-8, and polymers^30–32^. Those studies revealed that hydrophobic adsorbents potentially show the desired high selectivity for 1-butanol over water. However, the thorough investigation of adsorption behavior and adsorption mechanism of 1-butanol on adsorbents are rarely reported.

Thus, in the present work a series of HDPC materials were obtained by pyrolyzing hydrochar at different temperatures. The materials produced in this study were characterized and used for 1-butanol adsorption from aqueous solutions. The primary objective of this study is to investigate the main factors that affect 1-butanol adsorption and to explore the adsorption mechanism of 1-butanol on the HDPC materials.

## Experimental Section

### Materials and methods

The hydrochar was made from corncob. The HTC process can be briefly described as follows: A mixture of raw material (2.1 g) and deionized water (35 mL) were placed into an autoclave. The reactor was programmed to heat and then hold at a peak temperature (200 °C) for 24 h. After reaction, the hydrochar products were recovered by filtration and washed abundantly with distilled water and then dried at 100 °C for 12 h. Finally, the dried hydrochar sample was milled to pass a 0.25 mm sieve (60 mesh). The hydrochar was thermally treated at 350, 450, 550, 650 and 750 °C under a constant flow of N_2_ gas (200 mL min^−1^) for 2 h at heating rate of 4 °C min^−1^. The pyrolyzed samples hereafter were assigned as PC350, PC450, PC550, PC650, and PC750 according to the pyrolytic temperature.

### Characterization of Samples

The thermogravimetric analysis of hydrochar sample were performed in the temperature range of ambient to 800 °C in the presence of nitrogen at the heating rate of 10 °C min^−1^. Elemental (C, H, N) analyses were conducted using an EA 3000 elemental analyzer (Euro Vector). Ash content was measured by heating the sample at 550 °C for 6 h under an air atmosphere. The morphology of the samples was examined using scanning electron microscopy in Quanta 400 FEG. A Fourier-Elmer IR (Nicolet 6700) and X-ray photoeletron spectroscopy (XPS) were used to analyze the oxygen-containing functional groups and surface properties of samples. FTIR spectra were recorded in the 4000–400 cm^−1^ region. The XPS spectra were obtained using a Thermo Scientific ESCALAB 250ΧΙ instrument equipped with AI Kα monochromatized radiation with a 1486.6 eV X-ray source. The binding energies were referenced to the C1_S_ line at 284.8 eV.

Nitrogen (N_2_) adsorption-desorption isotherms and CO_2_ adsorption isotherms of the samples were measured using a autosorb iQ instrument (Quantachrome U.S.) at −196 °C and at 0 °C, respectively. The total surface area calculated using the Brunauer-Emmett-Teller (BET). The total pore volume was estimated from a single-N_2_ adsorbed point at a relative pressure of about 0.98. The micropore volume and external volume (meso- and macropores) were calculated by use of a t-plot method. Pore size distribution was evaluated using the Nonlinear Density Functional Theory (NLDFT) method based on N_2_ adsorption and desorption data. The narrow micropore volumes (pores with size < 0.7 nm) was determined using CO_2_ adsorption isotherms via Dubinin-Radushkevich equation.

### Batch sorption experiment

1-Butanol was selected as the model adsorbate. The adsorption equilibrium experiments were performed in 50 mL Erlenmeyer flasks which contain 0.25 g sample and 10 mL 1-butanol solution with different initial concentrations (C_O_; 2.5–25 g/L). The flasks were placed in the thermostatic shakers maintained at room temperature (25 ± 0.3 °C) for 24 h with agitation at 150 rpm. Blank solutions were also prepared and analyzed. The 1-butanol concentration was determined by a GC (7890 A, Agilent, USA) equipped with a 60 m × 0.25 mm × 0.25 um Agilent HP-INNOWAX 19091N-236 column. Adsorption capacities *Q*_*e*_ (mg/g) were calculated as following:

1 $$Qe=(C0-Ce)\cdot V/m$$

where *C*_0_ (g/L) is the initial concentration, and *Ce* (g/L) is the equilibrium 1-butanol concentration. *m* (g) and *V* (mL) respectively represent the dry mass of samples and the volume of solution.

The adsorption kinetics studies were performed in a manner similar to the sorption experiments described above. The effects of contact time on the 1-butanol adsorption of selected HDPC samples were investigated at various initial concentrations (5, 10 and 15 g/L) at room temperature. Samples were withdrawn at preset time intervals with a syringe, and then filtered through a 0.45 μm polytetrafluoroethylene (PTFE) membrane filter. Finally, the samples were analyzed by GC. The 1-butanol uptake at any time, *Q*_*t*_ (mg/g), was calculated using the equation:

2 $${Q}_{t}=({C}_{0}-{C}_{t})\cdot V/m$$

where *C*_*t*_ (g/L) is the 1-butonal concentration in the liquid phase at time *t* (min).

## Results and Discussion

### Characterization of hydrochar and HDPC

#### TG analysis of hydrochar

**Figure 1:**
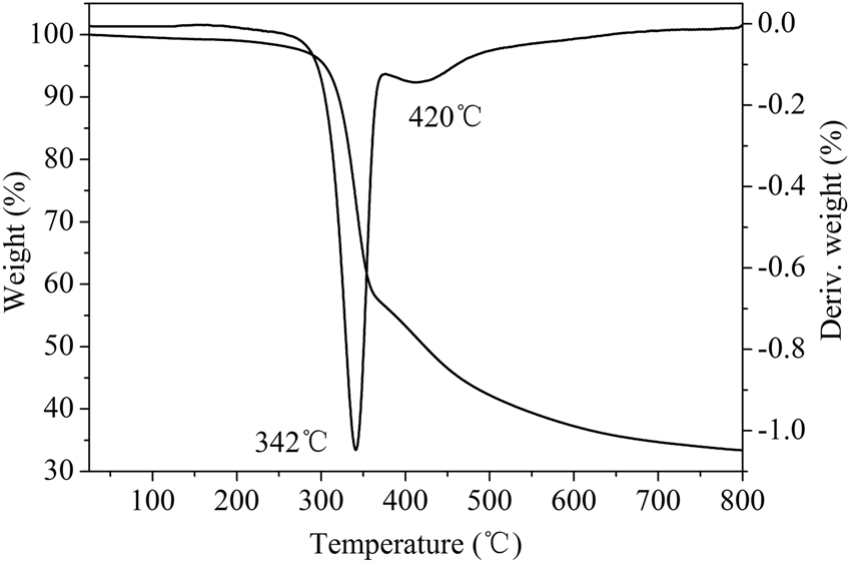
Pyrolysis curves of hydrochar.

The pyrolysis profile of hydrochar is shown in Fig. 1. The weight of hydrochar kept constant at low pyrolysis temperature (≤240 °C). Subsequently, the weight of hydrochar rapidly decreased with the further elevation of temperature, and finally the amount of residue left was around ~35%. The main DTG peak (342 °C) of hydrochar sample may be attributed to residual hemicellulose and cellulose decomposition and the weight loss at 420 °C resulted from the thermal degradation of lignin like compound or thermally produced carbonized/aromatic compounds^33^. Moreover, the lignin contributed to wide and flat DTG peak, due to its wide decomposition range. On the basis of pyrolysis profile, it can be seen that major physicochemical changes of the prepared hydrochar are accruing in the temperature range 350–750 °C. Thus, the pyrolysis temperature of hydrochar was chosen in the range of 350–750 °C as well^34^.

#### Proximate and elemental analysis

The proximate and elemental analysis of hydrochar and HDPC samples are presented in Table 1 and Fig. S1. Yield sharply decreased from 200 to 350 °C, due to the loss of residual hemicellulose and cellulose. The final yield of HDPC sample was 39.5%, which was in line with the TG measurement. The elemental analysis results demonstrate that carbon content advanced with increasing pyrolytic temperature, indicating high degree of carbonization at high temperature. Conversely, oxygen and hydrogen content decreased as the pyrolytic temperature increased. The O/C, (O + N)/C and H/C atomic ratios were calculated to evaluate the polarity and aromaticity, respectively, of the sample^35^. The lower atomic ratios in PC750 sample, showing that the sample was more aromatic and less hydrophilic compared to the hydrochar, due to higher extent of carbonization and loss of polar functional groups^36^. On the other hand, the higher atomic ratios for hydrochar and PC350 suggests that those samples contain numerous original residues and polar surface functional groups.

**Table 1:** Proximate and element analysis of hydrochar and HDPC samples.

Sample	Yield (%)	Ash(%)	C(%)	H(%)	O(%)	N(%)	H/C	O/C	(O + N)/C
HC	100	0.88	60.84	5.35	32.78	0.15	1.047	0.404	0.407
PC350	63.75	1.34	73.52	3.30	21.61	0.23	0.535	0.221	0.223
PC450	51.28	1.28	80.16	3.21	15.12	0.23	0.478	0.142	0.144
PC550	45.5	1.10	88.52	2.83	7.28	0.27	0.381	0.062	0.064
PC650	42.16	1.16	90.67	2.14	5.74	0.30	0.281	0.047	0.050
PC750	39.5	1.01	94.28	1.41	3.07	0.23	0.178	0.0 24	0.027

#### FTIR analysis

**Figure 2:**
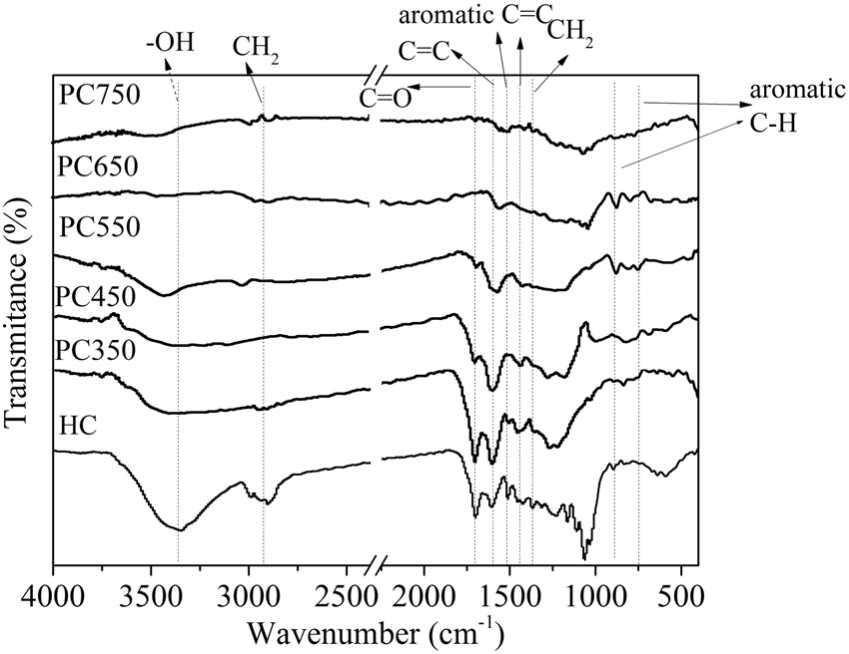
FTIR spectra of the hydrochar and HDPC samples.

The FTIR spectra of hydrochar and HDPC samples are shown in Fig. 2, which are consistent with the element analysis results. For the hydrochar, a broad absorption band between 3030 and 3680 cm^−1^ represents the stretching vibration of hydroxyl groups (O-H)^37^. The bands at 2920 and 1370 cm^−1^ are assigned to -CH_2_ stretching vibrations. The bands at 1700 and 1610 cm^−1^ can be ascribed to C=O and C=C stretching vibration, respectively. The peak of 1510 cm^−1^ represents the C=C ring stretching vibration of lignin and the band at the 1450 cm^−1^ is assigned to the aromatic ring stretching vibration. The band C-O-C (1160, 1110, 1065, and 1035 cm^−1^) represents O-containing function groups of the hydrochar. The peaks at 880 and 750 cm^−1^ are attributed to the aromatic CH out-of-plane deformation. For PC350, the bands at 3400 cm^−1^ (-OH), 2920 and 1370 cm^−1^ (-CH_2_-), 1510 cm^−1^ (C=C ring of lignin) and 1160–1035 cm^−1^ (C-O-C) are sharply decreased, whereas the bands at 1610 cm^−1^ (C=C) and 1700 cm^−1^ (ester C=O) are remained. It suggests that the dehydration, decarboxylation and demethylation reactions take place during the pyrolysis process. For PC450, the CH_2_ group and C=C ring of lignin completely diminished and the aromatic C=C stretching vibrations (1600 cm^−1^) increased^38,39^. At high pyrolytic temperature (550–750 °C), those residual bands are gradually decreased, and finally completely eliminated at 750 °C, which are similar for FTIR spectra of graphitic domains of amorphous carbon.

#### XPS analysis

**Figure 3:**
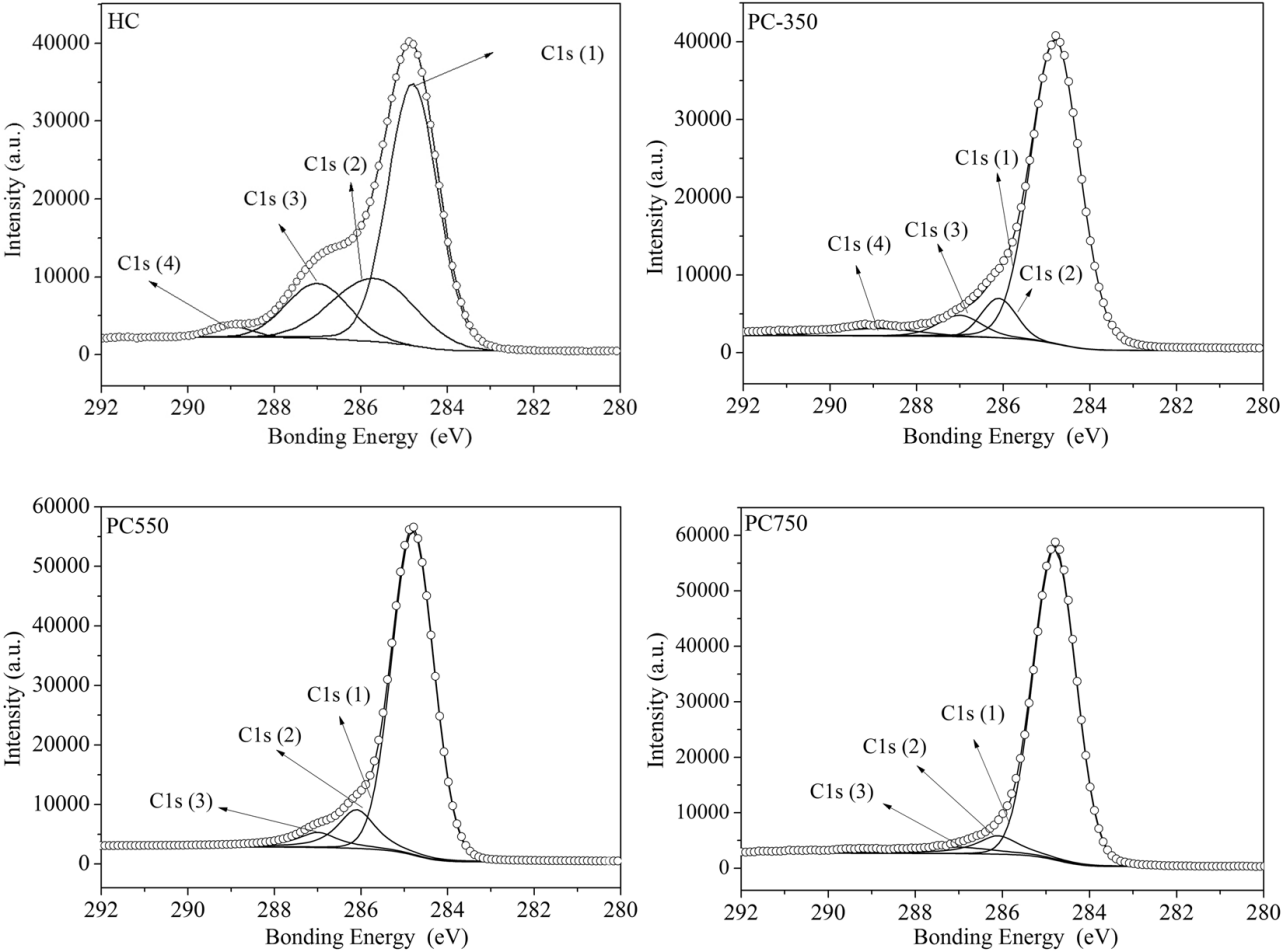
XPS data of hydrochar and selected HDPC samples.

X-ray photoeletron spectroscopy (XPS) was used to investigate surface polar/oxygen functional groups on the hydrochar and HDPC samples, and the data are shown in Fig. 3 and Table 2. For the hydrochar, the C1 _S_ spectrum was split into four signals at 284.8, 285.7, 287.0, and 288.5 eV. They are attributed to the graphitic carbon group (C1, C-C and C-H_X_), hydroxyl/ether group (C2, C-O-H and C-O-C), carbonyl/quinone groups (C3, C=O), and carboxylic/ester/lactone groups (C4, O=C-O)^22^. Differences from HC, the graphitic carbon group in PC350 have greatly increased, due to loss of O-containing function groups caused by thermal treatment, which was confirmed by FTIR. The C1 peak further elevated and the C4 peak completely disappeared upon increasing thermal temperature, indicating that the surfaces of sample were more aromatic and less hydrophilic^40^.

**Table 2:** Experimental C1(s), binding energy (eV) and chemical state assignments for hydrochar and selected HDPC samples.

sample	C1	C2	C3	C4
	C-C/C-H(%)	C-O(%)	C=O (%)	O=C-O (%)
HC	284.8/59.70	285.7/23.03	287.0/15.22	288.5/2.04
PC350	284.8/83.46	285.7/7.43	287.0/5.84	288.5/3.41
PC550	284.8/82.7	285.7/11.72	287.0/5.58	×
PC750	284.8/87.97	285.7/7.35	287.0/4.67	×

#### Morphology and structural properties of hydrochar and HDPC samples

Fig. S2 shows that the SEM images of hydrochar and HDPC samples. It can be seen that hydrochar consists of some microspheres and irregular structures which originated from cellulose and lignin without participating hydrothermal reaction. When further pyrolysis treatment in higher temperature, the morphology of HDPC samples is relatively stable and does not undergo great changes.

The porous textual properties of hydrochar and HDPC samples are analyzed by nitrogen sorption measurements. Table 3 exhibited that the surface area of hydrochar is extremely low (7.20 m^2^/g).This can be attributed to the dehydration, polymerization and condensation reaction in the hydrothermal process hinder the formation of pores. The surface area and the total pore volume of HDPC samples rapidly elevate from 16.71 m^2^/g and 0.052 m^3^/g at 350 °C to 502.45 m^2^/g and 0.279 m^3^/g at 550 °C. Finally, the surface area and total pore volume become relatively stable at higher temperature.

**Table 3:** Porous textual properties of hydrochar and HDPC samples.

Sample	S_BET_(m^2^/g)	S_mic_ (m^2^/g)	V_t_(cm^3^/g)	V_mic_(cm^3^/g)	n_CO2_(mmol/g)	V_DRCO2_
HC	7.20	0	0.047	0	0.540	0.061
PC350	16.71	0	0.052	0	1.419	0.097
PC450	227.18	167.21	0.15	0.068	1.990	0.157
PC550	502.45	383.61	0.279	0.155	2.490	0.199
PC650	517.55	476.27	0.237	0.184	2.770	0.217
PC750	502.38	462.15	0.238	0.179	2.920	0.232

The N_2_ adsorption-desorption isotherms and pore size distribution are presented in Figs S3 and S4, respectively. The N_2_ isotherms of hydrochar and PC350 are in line with type II nonporous materials in accordance with the IUPAC classification^41^. So, the low surface area of hydrochar and PC350 mainly result from interparticle voids. However, the other HDPC samples exhibit a type I isotherm, suggesting that the materials possess developed micropore structure. As shown in Fig. S3, the porosity of materials is mainly made up of micropores and the pore size distribution gradually decreased upon increasing treatment temperature.

CO_2_ adsorption isotherms of hydrochar and HDPC samples are shown in Fig. S5, with CO_2_ uptake and narrow micropore volume shown in Table 3. PC750 exhibit the largest CO_2_ uptake and narrow micropore volume, indicating that the structure of materials are significantly affected by the pyrolytic temperature^42^.

#### XRD analysis

**Figure 4:**
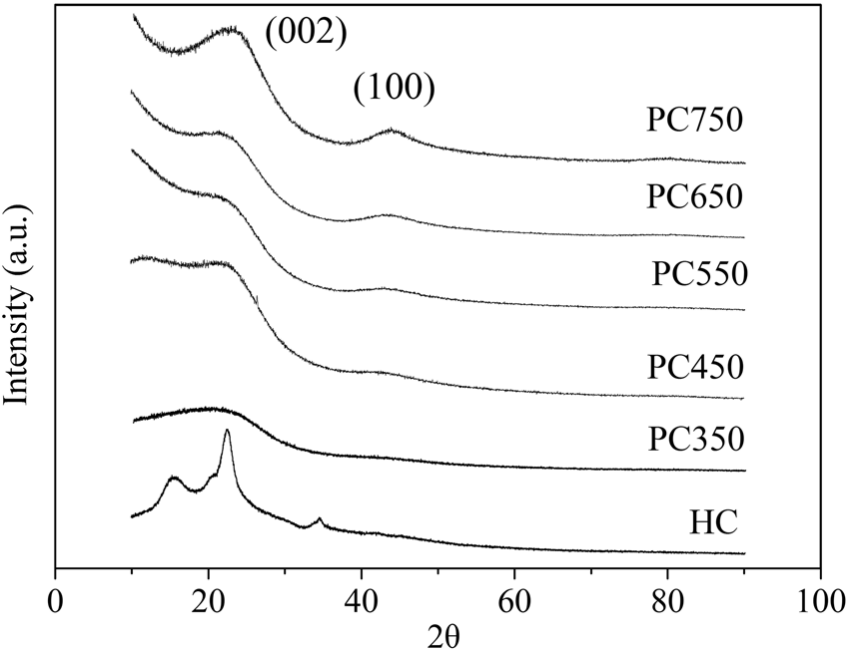
XRD profiles of hydrochar and HDPC samples at various thermal temperatures.

As shown in Fig. 4, the XRD pattern of hydrochar presents three peaks similar to characteristic peaks of cellulose, indicating that no all cellulose take part in hydrothermal reaction^43^. The three peaks completely disappeared in the PC350, indicating that the destruction of microcrystalline structure of cellulose. For PC450, two broad diffraction peak at around 2θ = 23° and 43° respectively, corresponding to (002) and (100) plane of turbostratic carbon^21^. The two peaks become sharper along with increasing thermal temperature, indicating that the atomic structural order improved and is attributed to the aromaticization and condensation of the materials^44^. The XRD pattern further confirmed the element analysis and FTIR results.

### Butanol adsorption

#### Adsorption isotherms

**Figure 5:**
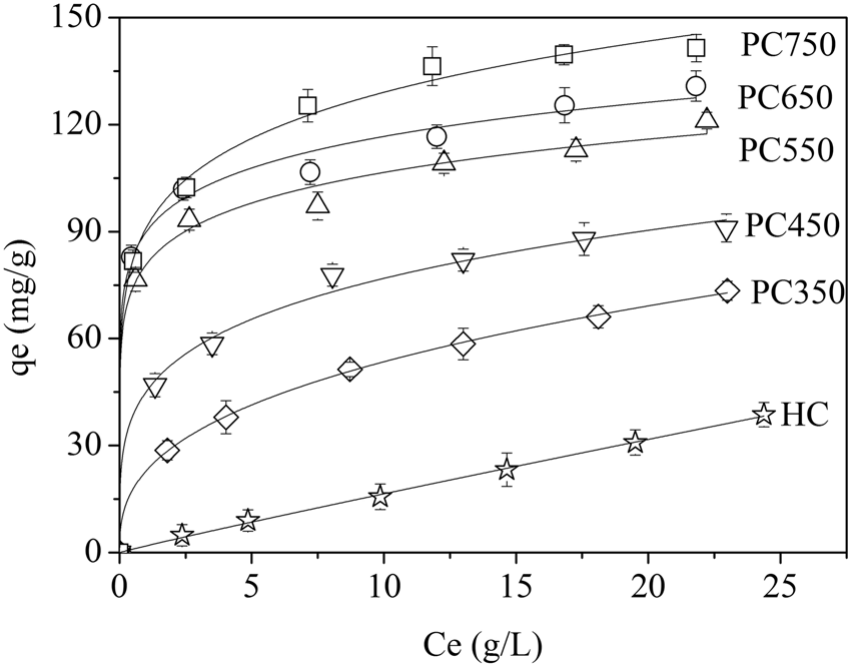
Freundlich isotherms of 1-butanol adsorption on hydrochar and HDPC samples.

Figures 5 and S6 exhibit the adsorption isotherms of 1-butanol (adsorbate) on the hydrochar and HDPC samples (adsorbent). It can be observed PC750 has the highest adsorption capacity to 1-butanol which reaches around 150 mg/g. Comparison with other adsorbents in terms of active carbons and polymer resins^45^, HDPC materials show good adsorption properties. Due to its renewable resources and low production costs, HDPC can be considered as a prior adsorbent for 1-butanol adsorption. The experimental data were fitted to the Langmuir and Freundlich models to evaluate the adsorption capacity of samples and interactions with adsorbent and adsorbate. Those models are described as follows:

3 $${\rm{L}}{\rm{a}}{\rm{n}}{\rm{g}}{\rm{m}}{\rm{u}}{\rm{i}}{\rm{r}}\,{\rm{m}}{\rm{o}}{\rm{d}}{\rm{e}}{\rm{l}}:{Q}_{e}=\frac{{Q}_{m}{K}_{L}{C}_{e}}{1+{K}_{L}{C}_{e}}$$

4 $${\rm{F}}{\rm{r}}{\rm{e}}{\rm{u}}{\rm{n}}{\rm{d}}{\rm{l}}{\rm{i}}{\rm{c}}{\rm{h}}\,{\rm{m}}{\rm{o}}{\rm{d}}{\rm{e}}{\rm{l}}:{Q}_{e}={{K}_{F}{C}_{e}}^{1/n}$$

where *Q*_*m*_ (mg/g) is the maximum adsorption capacity, *K*_*L*_ (L/mg) is the constant related to the free energy of adsorption; *K*_*F*_ ((mg/g)(L/g)^1/n^) is the Freundlich constant represented adsorption capacity of adsorbent, and *n* is the Freundlich exponent reflecting the adsorbate affinity towards the adsorbent. When the value of *n* higher than 1 (*1/n* < 1), indicating that a favorable adsorption process.

The characteristic parameters of Langmuir and Freundlich models are summarized in Table 4. Compared to the correlation coefficient *R* ^2^ of two models, Freundlich model better described the adsorption process. It demonstrates that the adsorption of hydrochar and HDPC samples mainly occur on heterogeneous surfaces^46^. The value of *K*_*F*_ in the Freundlich model increased with increasing thermal temperature, reflecting the adsorption capacity of samples elevated with pyrolysis temperature^47^. The *1/n* value is smaller than 1 in all cases, indicating the adsorption process is favorable^48^.

**Table 4:** Langmuir and Freundlich isotherm parameters for adsorption of 1-butanol onto hydrochar and HDPC samples.

Adsorbent	Freundlich			Langmuir		
	*K*_*F*_((mg/g)(L/g)^1/n^)	*1/n*	R^2^	*Q*_*m*_ (mg/g)	*K*_*L*_ (L/mg)	R^2^
HC	5.224	0.63	0.998	66.35	0.054	0.993
PC350	22.81	0.370	0.999	82.7	0.222	0.98
PC450	44.98	0.233	0.994	94.86	0.59	0.987
PC550	80.65	0.121	0.993	112.12	3.047	0.967
PC650	86.16	0.129	0.994	122.95	2.73	0.973
PC750	90.886	0.152	0.997	138.25	2.23	0.971

#### Analysis of interaction between material properties and 1-butanol adsorption

**Figure 6:**
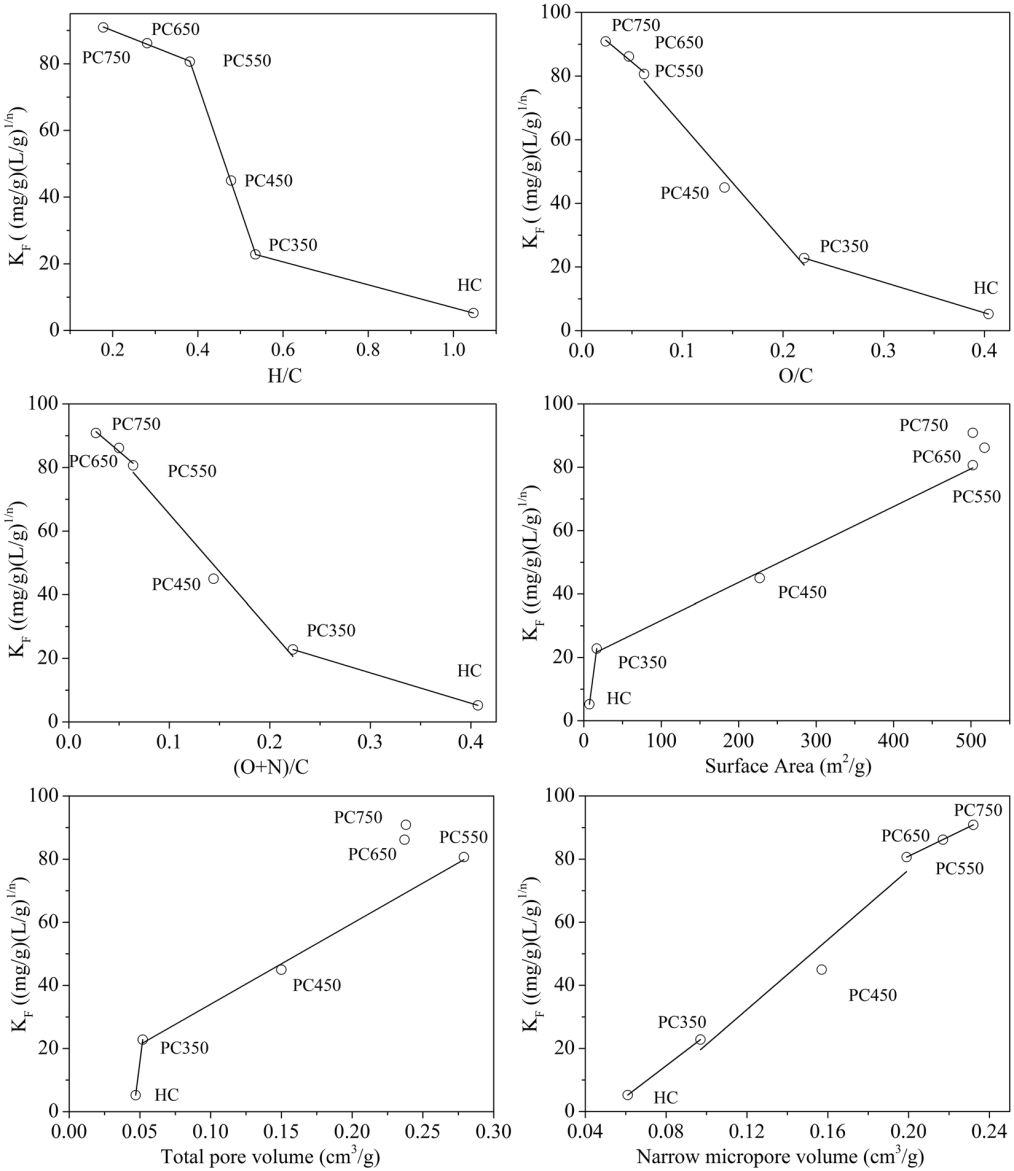
The percentage of adsorption of 1-butanol on the hydrochar and HDPC samples.

The adsorption of non-ionic organic compound, such as 1-butonal on carbonaceous materials depends on its physical and chemical properties^49^. In order to compare the adsorption performances of different samples, the *K*_*F*_ and *Q*_*e*12_ (extracted from the adsorption isotherms at a constant 1-butanol equilibrium concentration of 12 g/L) were correlated with various properties of the samples, including molar H/C, O/C, and (O + N)/C ratios, surface area, total pore volume, and narrow micropore volume (Figs 6 and S7). Generally, the value of *K*_*F*_ was negatively correlated with H/C (aromaticity index), O/C, and (N + O)/C atomic ratios (polarity index), and was positively correlated with the surface area, total pore volume, and narrow micropore volume (Fig. 6). Futhermore, the data was divided into three partitions to better state the correlation between sample properties and 1-butonal adsorption. The first portion is from hydrochar to PC350, the second portion is from PC350 to PC550, and the final portion between PC550 and PC750. Correlation results were listed in Tables S1 and S2.

With regard to the samples generated at different pyrolysis temperature, the reason causing the increase of adsorption capacity is distinctive. For the first portion, the *K*_*F*_ value of PC350 is 4.36 times higher than hydrochar, mainly due to the reduction of surface polar function groups (O/C and (O + N)/C). Hence, the hydrophilicity of surface O-containing functional groups hinder 1-butanol adsroption because of the enhanced competitive adsorption of water^50^. Relative to the surface functionality effect, the effect of surface area (porosity) of PC350 can be negligible in the 1-butanol adsorption. The *K*_*F*_ further rapidly increased from PC350 to PC550, can be attributed to the lost of O-containing functional groups and the considerable increase in surface area and total pore volume (pore-filling effect)^51^. The increase of temperature leads to the development of better pore structure resulting in high adsorption due to added accessibility of active binding sites for 1-butanol adsorption. Correspondingly, those properties of the samples have a good correlation with the *K*_*F*_ value (R^2^ > 0.982). For PC550-PC750, the *K*_*F*_ value slowly increased. It mainly ascribed to the promotion of aromaticity (H/C), hydrophobic (O/C) and narrow micropore volume. The increase of aromaticity and hydrophobic facilitated 1-butanol adsorption, suggesting that hydrophobic interactions and non-specific van der waals interactions occur during the adsorption process^22^. The same results had been reported by many other researchers. Anthony *et al*. have reported that 1-butanol preferentially adsorbed in hydrophobic surface of materials. Whereas, it is worth noticing that, when pyrolytic temperature is higher than 550 °C, the trend of surface area and total pore volume with the value of *K*_*F*_ are irregular and complex. The narrow pore volume plays a vital role in 1-butanol adsorption. The H/C, O/C ratio and narrow micropore volume were well fitted with the *K*_*F*_ value (R^2^ > 0.963).

The *Q*_*e12*_ value was also plotted against the properties of samples, as shown in Fig. S6. The conclusion is similar to the correlation analysis of *K*_*F*_ value and various properties of samples. Overall, the 1-butonal adsorption capacity is enhanced by high aromaticity, surface area, and pore volume and is unfavoured by the polar functional groups of materials. Simultaneously, at different pyrolysis temperature, the factors causing the increase of adsorption capacity of samples are variable.

#### Adsorption kinetic studies

The adsorption kinetics describes the dynamic adsorption process of the solute on the adsorbent. The kinetic parameters can help to predict the adsorption rate, which give critical information for designing and modeling the adsorption process^52^. In order to investigate the diffusion mechanisms, the kinetic adsorption data of 1-butanol on HDPC samples were analyzed using the intraparticle diffusion model. The model can be written by the following equation:

4 $${Q}_{t}={k}_{id}{t}^{1/2}+C$$

where *k*_*id*_ (mg/g min^1/2^) is the intraparticle diffusion rate constant, and *C* is the intercept which represents the boundary layer thickness. If the intraparticle diffusion is the sole rate-limiting step, the plot of *Q*_*t*_ versus *t* ^*1/2*^ should be linear and passes through the origin. Otherwise, some other mechanism may be also involved and to influence the adsorption process^53^.

**Figure 7:**
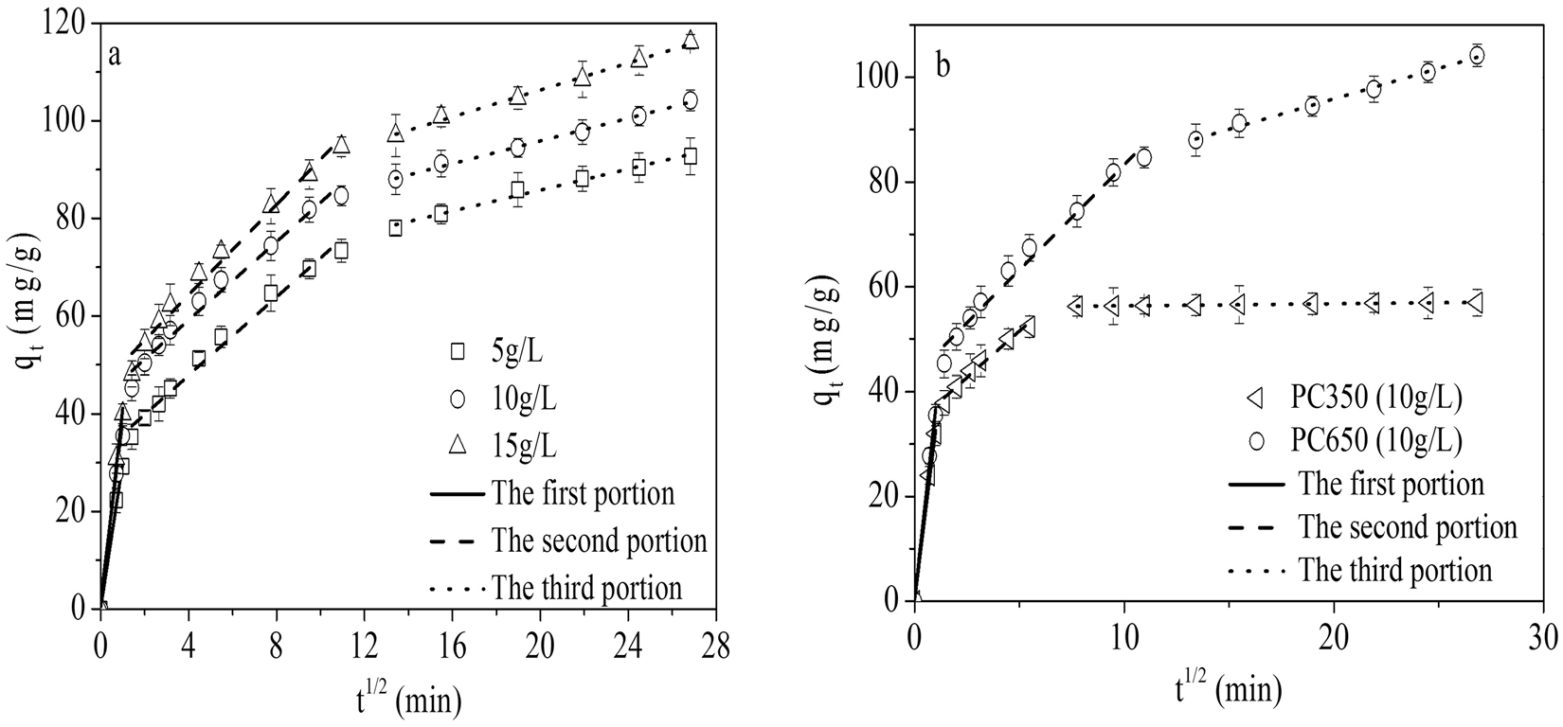
Plot of intraparticle diffusion model for adsorption of 1-butanol on selected samples at different initial 1-butanol concentration.

The kinetic adsorption curves for PC650 obtained at different initial 1-butanol concentrations (Fig. 7a) showed tri-linearity, indicating that three steps took place in the adsorption process. The first sharper portion represents the external surface adsorption or the boundary layer diffusion of solute molecule. The second portion illustrates the gradual adsorption stage, where intraparticle diffusion is rate-limiting (diffusion in mesopores). The final portion is owed to the equilibrium adsorption stage, where intraparticle diffusion starts to slow down due to the low 1-butanol concentration in solution (diffusion in micropores). Among these three processes, however, only one is the rate limiting step in any particular time range.

The values of kid and correlation coefficient R2 are shown in Table 5. The kid value represents the rate of adsorption. A low kid value corresponds to a slow adsorption process. It can be observed that the kid1 and kid2 values are higher than kid3 value, indicating that the diffusion process of 1-butanol molecules from bulk phase to the exterior surface of adsorbent and inside the mesopores considerably rapid. Conversely, the third portion contains the lowest rate constant and the longest contact time. It illustrates that the diffusion of 1-butanol molecules in micropores is the rate-limiting step over the whole adsorption process^54^. Moreover, the adsorption dynamic curve of PC350 at initial 1-butanol of 10 g/L was performed and the result is shown in Fig. 7b and Table S3. It can be observed it takes less than 10 min to reach adsorption equilibrium. Comparison to the curve of PC650, the adsorption capacity of PC350 is lower and the adsorption equilibrium time is faster, which is due to a great discrepancy in the pore structure of these two samples. The dynamics experiment further confirmed that the pores play a vital role in the 1-butonal adsorption.

**Table 5:** Intraparticle diffusion model constants at different initial 1-butanol concentration, T = (25 ± 0.3) °C.

Concentration (g/L)		5	10	15
Parameters	*k*_*id1*_(mg min^−1^)	30.003	36.753	40.706
	R^2^	0.996	0.993	0.995
	*k*_*id2*_(mg min^−1^)	3.859	4.019	4.633
	R^2^	0.989	0.981	0.983
	*k*_*id3*_(mg min^−1^)	1.077	1.164	1.38
	R^2^	0.986	0.996	0.996

## Conclusions

The physical and chemical properties of HDPC samples, significantly influenced by the pyrolytic temperature, play a crucial role in the adsorption of 1-butanol from aqueous solution. In general, the 1-butanol adsorption capacity is enhanced by high aromacity, hydrophobicity and porosity (surface area, total pore volume and narrow micropore volume) and low surface O-containing functional groups of samples. However, at different pyrolysis temperature, the factors causing the increase of 1-butanol adsorption capacity is difference. The lost of surface functional groups dominant the increase of 1-butanol adsorption at low thermal temperature (<350 °C). Pyrolysis temperature between 350 °C and 550 °C, the 1-butanol adsorption increased with a decreased in polarity and the considerable increase in surface area and pore volume of HDPC samples. At higher pyrolysis temperature, the 1-butanol adsorption elevated, mainly due to the increase in aromaticity, hydrophobicity and narrow micropore volume. Simultaneously, intraparticle diffusion model was used to study the adsorption mechanism of 1-butanol on the selected HDPC samples, and the diffusion in micropores was considered as the rate-controlling step over the whole steps.

## Electronic supplementary material

SUPLEMENTARY MATERIAL
