# Supplementary material for: Bio-butanol sorption performance on novel porous-carbon adsorbents from corncob prepared via hydrothermal carbonization and post-pyrolysis method
Source: Sci Rep. 2017 Sep 18;7:11753. doi: 10.1038/s41598-017-12062-7 (PMC5603594; doi:10.1038/s41598-017-12062-7)
Supplement: Supplementary file 1 — SUPLEMENTARY MATERIAL [file 41598_2017_12062_MOESM1_ESM.pdf]

SUPLEMENTARY MATERIAL

Bio-butanol sorption performance on novel porous-carbon adsorbents from corncob prepared via hydrothermal carbonization and post-pyrolysis method

Mengjun Han[a,b,c], Kangkang Jiang[a,b,c], Pengfei Jiao[a,b,c], Yingchun Ji[a,b,c], Jingwei Zhou[a,b,c], Wei Zhuang[a,b,c], Yong Chen[a,b,c], Dong Liu[a,b,c], Chenjie Zhu[a,b,c], Xiaochun Chen[a,b,c], Hanjie Ying[a,b,c,d,*], Jinglan Wu[a,b,c,*]

[a]*College of Biotechnology and Pharmaceutical Engineering, Nanjing Tech University, Nanjing, China*

[b]*National Engineering Technique Research Center for Biotechnology, Nanjing, China*

[c]*Jiangsu National Synergetic Innovation Center for Advanced Materials, Nanjing, China*

[d]*State Key Laboratory of Materials-Oriented Chemical Engineering, Nanjing, China*

[*]Corresponding Author.

Jinglan Wu: E-mail: wujinglan@njtech.edu.cn; Address: College of Biotechnology and Pharmaceutical Engineering, Nanjing Tech University, Xin Mofan Road 5, Nanjing 210009, China. (Tel. +86-25-86990001; Fax: +86-25-58139389);

Hanjie Ying: E-mail: yinghanjie@njtech.edu.cn; Address: College of Biotechnology and Pharmaceutical Engineering, Nanjing Tech University, Xin Mofan Road 5, Nanjing 210009, China. (Tel. +86-25-86990001; Fax: +86-25-58139389)

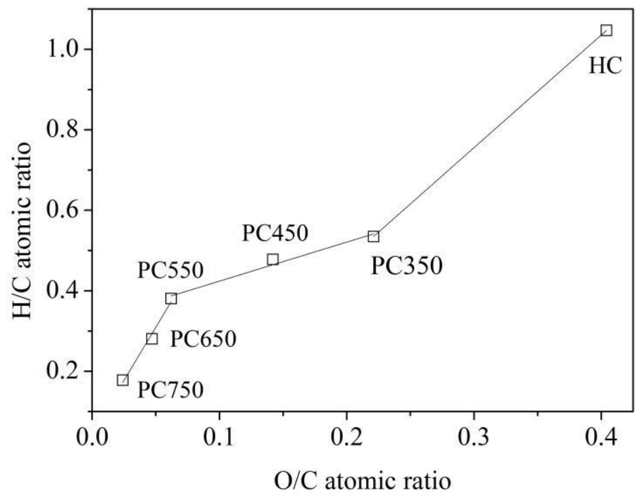

Fig.S1. H/C ratio versus O/C ratio of hydrochar and HDPC samples obtained at different thermal temperature.

For simplicity, the trend in elemental change of hydrochar and HDPC samples is linearly plotted. The line of H/C versus O/C below 350 ℃ appeared a moderate slope, and then it turned with a smaller slope within 350 and 550 ℃. Ultimately, the slope become larger between 550 and 750 ℃. Dehydration and deploymerization of hydrochar to release CO_2, CH_4 and steam, were responsible for a moderate slope below 350 ℃[1-3]. A smaller slope between 350 and 550 ℃ indicated that the decarboxylation, decarbonylation and deetherification reaction (loss of CO_2, CO) was involved during this period. A larger slope above 550 ℃ was mainly due to the release of CH_4 and H_2 (demethylation and aromatization)[4].

In general, the value of H/C and O/C ratio decreased with increasing thermal temperature, suggesting richer carbon products are formed[5,6].

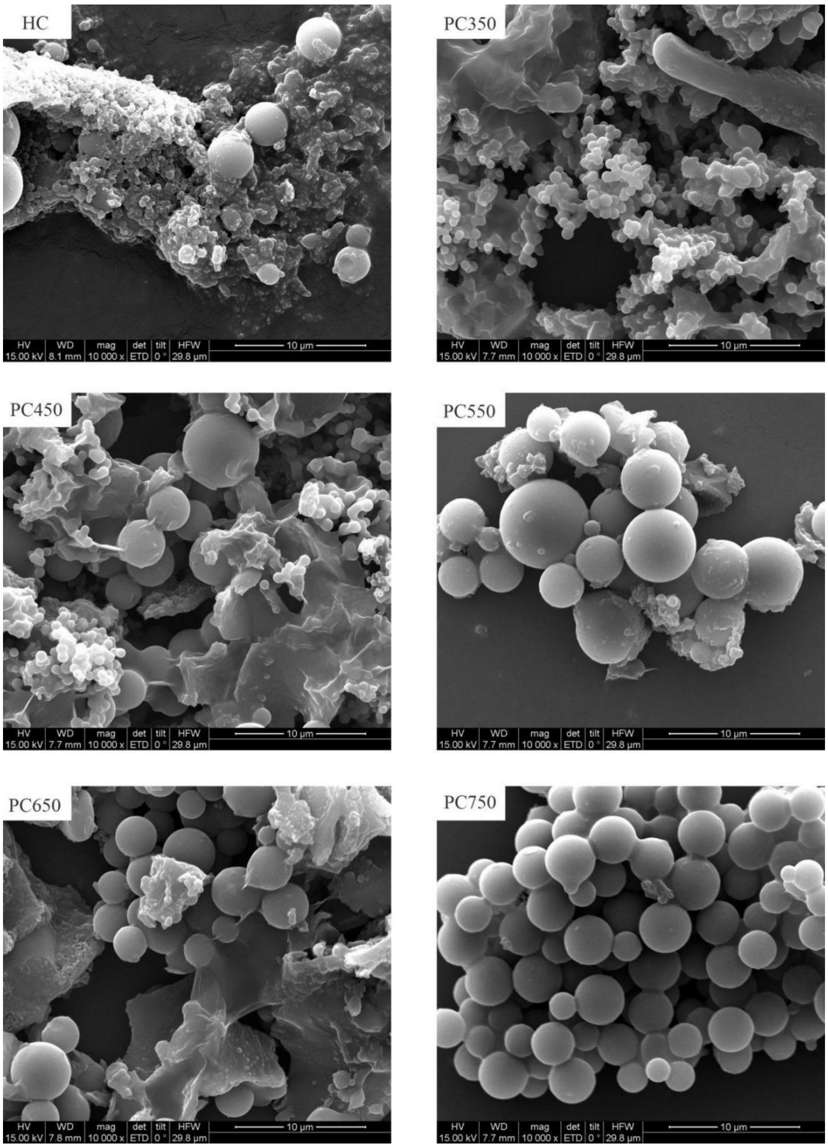

Fig.S2. SEM images of hydrochar and HDPC samples

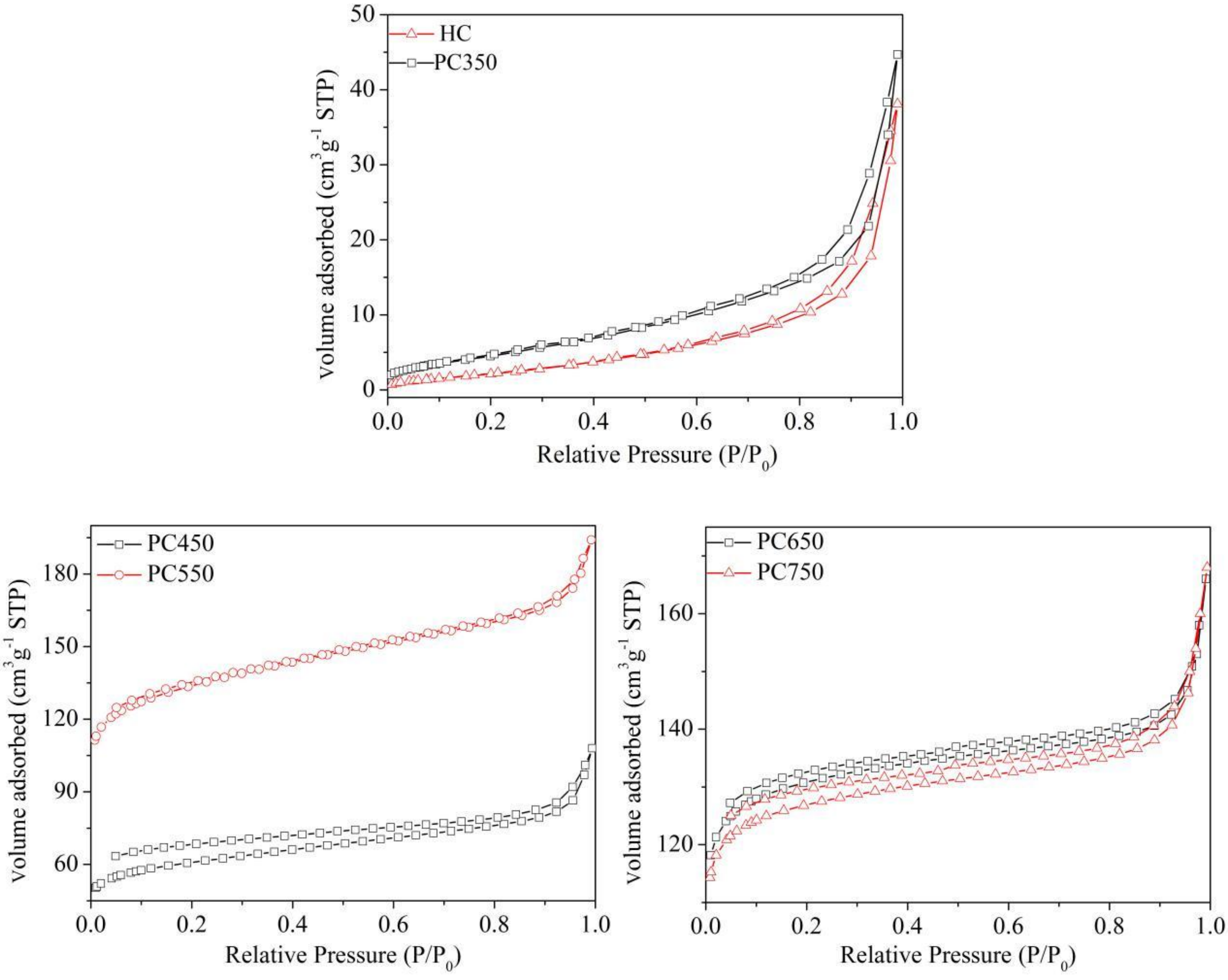

Fig.S3. Nitrogen adsorption-desorption isotherms of hydrochar and HDPC samples

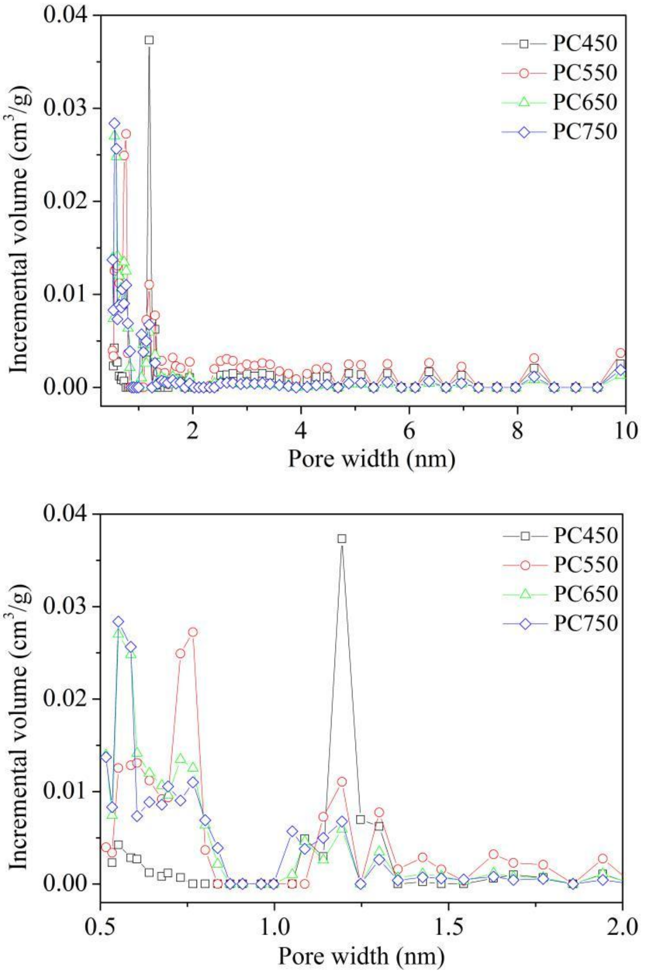

Fig.S4. Pore size distribution of hydrochar and HDPC samples

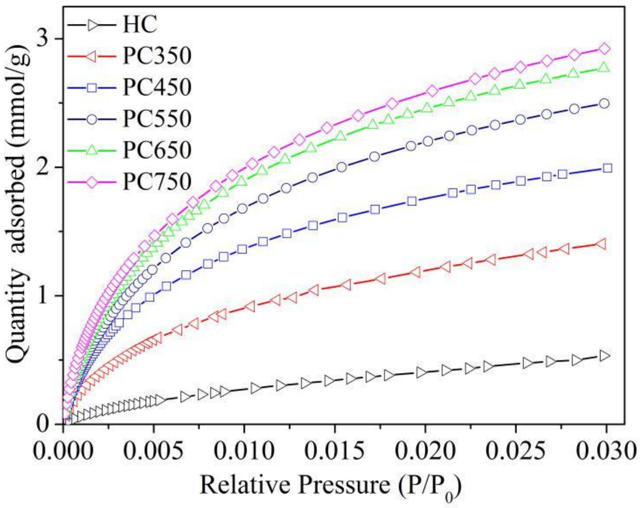

Fig.S5. Carbon dioxide adsorption isotherms (0 ℃) of hydrochar and HDPC samples

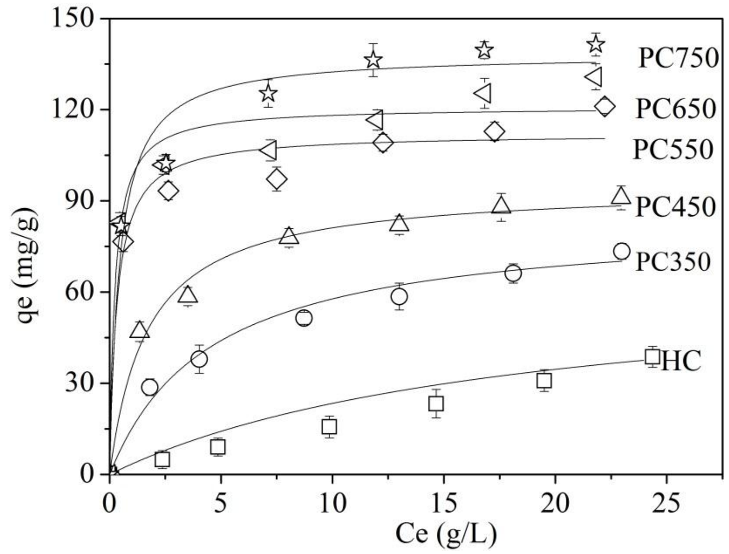

Fig.S6. Langmuir isotherms of 1-butanol adsorption on hydrochar and HDPC samples.

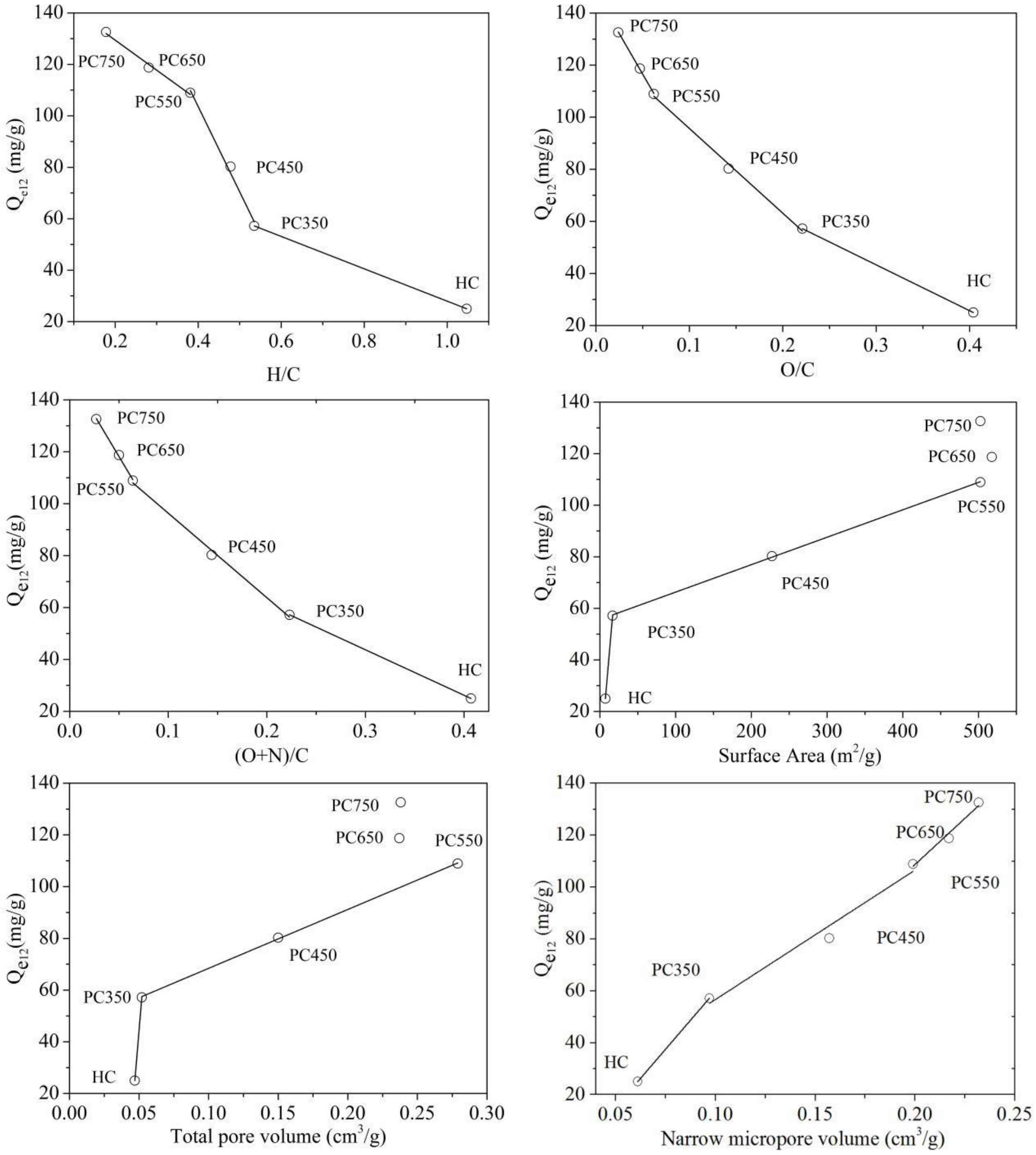

Fig.S7. 1-butanoldsorption capacity of hydrochar and HDPC at 12 g L^{-1} 1-butanol equilibrium concentration as a function of the property of hydrochar and HDPC samples

Table S1. The linear correlation between 1-butanoladsorption capacity (K_F)and the property of hydrochar and HDPC samples at different partitions

	the first portion (200-350 ℃)	R^2
H/C-K_F	y = -34.329x + 41.171	- -
O/C-K_F	y = -95.716x + 43.934	- -
(O+N)/C-K_F	y = -96.013x + 44.257	- -
surface area-K_F	y = 1.8496x - 8.0974	- -
Total pore volume-K_F	y = 3664.6x - 167.75	- -
Narrow micropore volume-K_F	y = 586.33x - 12.37	- -
	the second portion (350-550 ℃)	R^2
H/C-K_F	y = -50.388x + 100.02	0.997
O/C-K_F	y = -363.94x + 100.91	0.983
(O+N)/C-K_F	y = -363.73x + 101.82	0.982
surface area-K_F	y = 0.11958x + 19.727	0.997
Total pore volume-K_F	y= y = 255.87x + 8.4518	0.997
Narrow micropore volume-K_F	y = 383.24x - 3.6662	0.877
	the third portion (550-750 ℃)	R^2
H/C-K_F	y = -376.37x + 224.38	0.999
O/C-K_F	y = -268.03x + 97.839	0.968
(O+N)/C-K_F	y = -263.21x + 98.286	0.963
surface area-K_F	×	×
Total pore volume-K_F	×	×
Narrow micropore volume-K_F	y = 310.18x + 18.894	0.999

Table S2. The linear correlation between adsorption capacity of hydrochar and HDPC at 12 g L^{-1} 1-butanol equilibrium concentration and the property of materials at different partitions

	the first portion (200-350 ℃)	R^2
H/C-Q_{e12}	y = -62.881x + 90.833	- -
O/C-Q_{e12}	y = -175.33x + 95.894	- -
(O+N)/C-Q_{e12}	y = -175.87x + 96.486	- -
surface area-Q_{e12}	y = 3.388x + 0.58631	- -
Total pore volume-Q_{e12}	y = 6712.5x - 291.85	- -
Narrow micropore volume-Q_{e12}	y = 1074x - 7.24	- -
	the second portion (350-550 ℃)	R^2
H/C-Q_{e12}	y = -333.13x + 236.94	0.993
O/C-Q_{e12}	y = -325.47x + 128.12	0.996
(O+N)/C-Q_{e12}	y = -325.3x + 128.94	0.996
surface area-Q_{e12}	y = 0.10637x + 55.662	0.999
Total pore volume-Q_{e12}	y = 227.58x + 45.637	0.999
Narrow micropore volume-Q_{e12}	y = 348.59x + 33.787	0.919
	the third portion (550-750 ℃)	R^2
H/C-Q_{e12}	y = -116.55x + 152.74	0.991
O/C-Q_{e12}	y = -631.7x + 148.23	0.999
(O+N)/C-Q_{e12}	y = -621.5x + 149.34	0.998
surface area-Q_{e12}	×	×
Total pore volume-Q_{e12}	×	×
Narrow micropore volume-Q_{e12}	y = 711.4x - 33.588	0.977

Table S3. Intraparticle diffusion model constants for 1-butanol adsorption onto PC350 and PC650 , T= (25±0.3) ℃.

sample		PC350	PC650
Parameters	K_{id1}(mg min^{-1})	32.6	36.753
	R^2	0.993	0.993
	K_{id2}(mg min^{-1})	3.52	4.254
	R^2	0.979	0.988
	K_{id3}(mg min^{-1})	0.023	0.394
	R^2	0.981	0.993

1 Keiluweit, M., Nico, P. S., Johnson, M. G. & Kleber, M. Dynamic molecular structure of plant biomass-derived black carbon (biochar). *Environmental Science & Technology* **44**, 1247-1253 (2010).

2 Chen, Z., Chen, B. & Chiou, C. T. Fast and Slow Rates of Naphthalene Sorption to Biochars Produced at Different Temperatures. *Environmental Science & Technology* **46**, 11104-11111 (2012).

3 Kim, W. K., Shim, T., Kim, Y. S., Hyun, S. & Ryu, C. Characterization of cadmium removal from aqueous solution by biochar produced from a giant Miscanthus at different pyrolytic temperatures. *Bioresource Technology* **138**, 266 (2013).

4 Chen, Z., Chen, B., Zhou, D. & Chen, W. Bisolute Sorption and Thermodynamic Behavior of Organic Pollutants to Biomass-derived Biochars at Two Pyrolytic Temperatures. *Environmental Science & Technology* **46**, 12476 (2012).

5 Zhu, X. *et al.* Role of Hydrochar Properties on the Porosity of Hydrochar-based Porous Carbon for Their Sustainable Application. *Acs Sustainable Chemistry & Engineering* **3**, 150413095859005 (2015).

6 Chun, Y., Sheng, G., Chiou, C. T. & Xing, B. Compositions and sorptive properties of crop residue-derived chars. *Environmental Science & Technology* **38**, págs. 4649-4655 (2004).
